# The Co‐Production, Pilot and Qualitative Evaluation of a Cancer Prevention Programme With High‐Risk Women Delivered on Group Walks by Cancer Champions: Shoulder to Shoulder, Walk and Talk

**DOI:** 10.1111/hex.14175

**Published:** 2024-08-08

**Authors:** Sarah Hanson, Wendy Hardeman

**Affiliations:** ^1^ School of Health Sciences University of East Anglia Norwich UK

**Keywords:** cancer prevention, co‐production, health inequalities, motivational interviewing

## Abstract

**Objectives:**

Women in the criminal justice system and women who have been subject to domestic abuse are at high risk of cancer but underrepresented in health promotion research. We aimed to co‐produce, pilot and evaluate a health promoting programme delivered on group walks.

**Design:**

A programme co‐produced by women, based on motivational interviewing, created the opportunity for supportive conversations about cancer prevention.

**Methods:**

Programme development in two workshops with women with lived experience using authentic vignettes to prompt help‐seeking conversations. A small pilot and a qualitative evaluation was done using framework analysis.

**Results:**

The programme appeared acceptable to women and the walk leaders. Women felt included and found it a safe space for sensitive conversations. They appeared empowered and more confident to seek help. Walk leaders expressed confidence in delivering this informal programme, which used prompts rather than delivering didactic training.

**Conclusion:**

Cancer prevention for high‐risk groups can be delivered in a personalised and novel way by creating informal opportunities for supportive conversations about cancer prevention. Careful co‐production of the programme of walks with women, using scenarios and quotes that were authentic vignettes, ensured that these came directly from the women's lived experience and enabled women to talk about change. Our findings indicate that this approach was practical, relevant and acceptable to them with some evidence of women feeling empowered to make informed decisions about their health. We recommend that future cancer prevention programmes for underrepresented groups take an asset‐based approach by utilising pre‐existing community organisations to increase reach and sustainability.

**Patient and Public Involvement:**

Women with lived experience co‐designed and tested the programme. Provisional findings were fed back to the women and the women's organisation that partnered with this research.

## 1 Introduction

Many interventions designed to prevent cancer, such as screening [1] and raising awareness of symptoms [2], fail to reach those with the greatest health needs, disadvantaged and vulnerable groups. This leads to socioeconomic disparities in cancer prevention for more socio‐economically deprived groups [3, 4, 5, 6].

Cancer incidence rates in England are higher in the most deprived quintile compared with the least for most cancer types (2013–2017). Globally, breast cancer is the most commonly diagnosed cancer, with 2.3 million cases in 2020, and the most common cause of cancer death in women [7]. Screening for breast and cervical cancer is strongly related with a reduction in cancer mortality. Globally, where a woman lives and her socioeconomic status largely determine women's cancer diagnosis and survival rates [8]. An analysis of breast and cervical cancer screening in 30 European countries found extreme under‐screening concentrated among lower income quintiles [9]. An analysis of 20 years of the breast screening service in the United Kingdom found a breast cancer mortality reduction of 60% in those who had attended in the last 3 years [10]. A recent systematic review found a reduction in breast cancer mortality in attenders versus nonattenders for breast screening in Europe, ranging between 33% and 43% (Northern Europe), 43%–45% (Southern Europe) and 12%–58% (Western Europe) [11]. In Nordic countries, projected incidence rates for cervical cancer in 2006–2010 would have been between three and five times higher than observed rates with over 60 000 cases likely prevented by the introduction of screening in the late 1960s [12].

Many studies point to the role of health literacy in the effectiveness of cancer screening programmes. People with higher levels of education are more likely to participate in screening for cervical, breast and colorectal cancer [13, 14, 15].

Cancer symptom awareness and cancer survival are positively associated [16]. Advanced stage of cancer at presentation is too often attributed to low socioeconomic status [17, 18]. This may be due to a failure to be able to interpret symptoms [2], evidence in the United Kingdom that people are worried about wasting doctor's time [19], poor understanding of risk factors, for example in cervical cancer [20], and inadequate health literacy cited as a major factor [21].

Understanding health information is important to help members of the public make appropriate decisions about their health. The conceptualisation of health literacy is at its simplest, functional skills to understand basic health messages [22]. More recently, the focus is on higher level skills to enable the use of information for prevention and self‐management as well as building empowerment and control and building community capacity for social action [23]. People are disadvantaged if they do not have the capacity to obtain, process and understand cancer information and services to make appropriate healthcare decisions that may limit their understanding of cancer screening and symptoms of cancer, adversely affecting their stage at diagnosis [21]. This may negatively impact psychological capability to perform cancer prevention behaviours, such as attending screening [24]. Despite the fact that health literacy is increasingly recognised as a critical factor in cancer prevention, research on successful methods for supporting those who have limited health literacy is needed to enable greater informed decision‐making on screening and reporting symptoms.

Women who are victims of domestic abuse and female offenders are at particularly high risk of cancer through multiple risk factors such as socioeconomic disadvantage, personal circumstance, addictive behaviours and low screening uptake [3, 25, 26, 27, 28, 29]. For example, 57% of women in prisons report being victims of domestic violence with over half reporting experiences of emotional, physical or sexual abuse as a child [30]. Both abuse and a parent in prison are considered ‘adverse childhood experiences’, and these are linked to early morbidity, chronic disease and cancer [31, 32]. Our previous qualitative research with women in the criminal justice system and women who had suffered intimate partner abuse aimed to better understand cancer risk from their perspective [33]. Our findings indicated that preventative health, cancer screening and early symptom awareness were lost in the whirlwind of everyday life. Women's lives were highly complex (rather than the pejorative ‘chaotic behaviours’). They had long histories of being let down by state services and people they should have been able to trust.

There is a need for sustainable and nontraditional approaches to engage women at high risk of cancer. Group walks are a promising and novel approach to engage women and have supportive conversations about cancer prevention. Our previous research in areas of high deprivation has shown the supportive nature of walking groups and that health information is often shared in an informal way in the form of anecdotes and experiences [25]. This is in addition to the multiple psychological and physiological health benefits of group walking [34].

We therefore worked with women, in their own space, in two women's centres to co‐produce a health promotion programme that worked for, and was relevant, to them. ‘Shoulder to Shoulder, Walk and Talk’ was conceptualised as a novel way to improve health literacy around cancer by talking about cancer screening and symptom awareness on group walks. Based on the COM‐B model of behaviour change [24], the logic was that walking groups could provide the social and physical opportunity to guide conversations about cancer prevention to promote intrinsic motivation, capability and confidence to make changes and informed choices. In addition, social and peer support might provide a meaningful way to promote health to highly vulnerable and marginalised populations [33]. The framework for the group walks programme was based on the principles of motivational interviewing to support self‐efficacy for change [35] with a focus on change talk [36] to support behaviour change around help seeking, reducing embarrassment in talking to health professionals and increasing confidence in attending screening programmes. Motivational interviewing uses change talk which is a way of having healthy conversations to support people in making decisions about their own lives. It does not tell a person what to do because it remains their freedom to make their own decisions. Rather, the emphasis is on active listening and establishing a good relationship of trust and truthfulness.

The aim therefore was to co‐produce and conduct a small pilot and qualitative evaluation of a novel programme. Delivered during a women's walking group, walk leaders promoted and shared health information about cancer screening and early symptom awareness to women at high risk of cancer in a supportive and trusted way, tailored to their health literacy needs.

## 2 Methods

Ethical approval was gained from HMPPS and institutional approval from the University of East Anglia, England, Ref 2018/19‐099 to co‐produce the ‘Shoulder to Shoulder, Walk and Talk programme’, pilot and qualitatively evaluate it. The women involved in this study attended women's centres in two places in the East of England in areas of high socioeconomic deprivation. The centres supported women serving community sentences in the English justice system and women who were victims of domestic abuse. The Cancer Champion Walk Leaders and the women attending the centres were invited to join the research, including the development of the programme and its qualitative evaluation using observation and interview methods. All those approached gave informed consent.

The methods are split into three sections: co‐production, piloting and evaluation.

### 2.1 The Co‐Production of the Intervention With the Women

From June 2018, two local Women Centres started ‘Walk and Talk’ groups as a way of sharing experiences in an enjoyable and supportive environment. The women who attended the centres were enroled in community justice and domestic abuse programmes and support back into work. The lead author (a white woman, postdoctoral community health researcher and registered nurse) completed other research with the centres [33] in 2016–2017 and maintained contact, supporting the development of their walks programme and occasionally walking alongside to help maintain long‐term trust and familiarity with the women.

We needed to understand what would be practical, relevant and acceptable in terms of the programme's format and what would be desirable qualities in potential Cancer Champion Walk Leaders. Using an outline programme that the researchers had tentatively developed and vignettes, in the form of authentic quotes from our previous work as discussion items [33], SH ran three co‐production workshops, with a researcher as support and note taker. An example vignette was this, which used an authentic quote: ‘One lady said to us, “For me to ask for help, even from my GP it takes a lot, it takes a lot for me to do that because of my past and people letting me down it's like “Ok if I open up and speak to people then I'm gonna be let down again”. Do you have views on that/how do you feel about that lady's story?”’. All vignettes were checked with the women, and we had long conversations about these as they saw them as authentic and elicited appropriate responses. In total, seven women with lived experience of domestic abuse and who were subject to the criminal justice system (custodial and noncustodial) were involved. Each workshop lasted approximately 90 min where we co‐developed the ‘Walk and Talk’ programme using the logic, experience and skills of the women who are ‘experts by experience’ [21, 37]. During the three workshops, we experimented with giving and receiving information on a group walk, in a quiet, but public place, and women said they felt comfortable with this. We made many edits to the outline programme: for example, women asked that we specifically discuss how women talk about their body parts. We therefore added a section entitled, ‘The embarrassing bit—what do women call their bits?’ and also a website on breast self‐examination that they had heard of called ‘Coppafeel’ (see link:https://coppafeel.org/). We also asked them how we could use the ‘readiness to change ruler’ (a key aspect of motivational interviewing that focuses on what people can do, on a 1–10 scale, rather than what they cannot do). They suggested making a laminated ruler and making sure that we focus on the fact that the numbers do not matter, rather that it is paramount to focus on what confidence and motivation the women do have to change. Walk leaders were trained in using the ruler. Additionally, a major focus of the workshop was the skills and qualities of being a ‘Cancer champion walk leader’. This was translated into the training by incorporating the following examples of skills and qualities: ‘Genuinely cares about the health and well‐being of the women in the group and promoting cancer prevention’; ‘Is open‐minded and prepared to try things in new ways’ and ‘Shows humour and a light‐hearted approach as appropriate’. In the event, two women volunteered to be walk leaders. This was determined by the women's centre and was not in the control of the researchers.

The output of the co‐production workshops was a walks programme to be delivered over approximately 5 weeks. An outline of the programme is in File S2, and a full copy of the programme is available at reasonable request.

### 2.2 Piloting

The piloting of the programme, which included women in the justice system, started in January 2020 with four ‘Walk and Talk’ group walks delivered by a trained Cancer Champion Walk Leader (Walk Leader 1) with three to five women and ceased in February 2020 due to COVID‐19. They were not contacted for evaluation of the scheme as by 2021 they were no longer in contact with the women's centre. The group walks and the research were re‐started in September 2021. In this second iteration, six walks were delivered (by Walk Leader 2) with six new women from the women's centre, who attended between three and six times. The second iteration did not include women in the justice system due to the profound repercussions of CoVID‐19 on community organisations and restrictions on meeting as a group.

### 2.3 Evaluation of the Programme

The research was verbally promoted by the manager of the centres who also issued participant information sheets. Consent forms were signed by all participants before joining the walks and before the evaluation.

The programme was piloted twice by two different walk leaders and evaluated using observation sheets. Two interviews and one focus group after the pilot were completed (sample questions in File S1). A standardised observation sheet (File S4) was developed to enable structured fieldnotes to be recorded. These were completed by the researcher after the walk. They included notes on the components of the intervention delivered (e.g., cancer screening discussed), any other topics of conversation (general and health) as well as the general atmosphere and dynamic. The walk leaders were interviewed to gain insights into the training and delivery of the programme.

The focus group with women from the second iteration of the walking group explored their views on the acceptability of the programme and help‐seeking behaviours. We took an interpretivist approach as we aimed to ask searching questions to understand women's subjective understandings, perceptions and social meanings of the appropriateness of giving and receiving health information in a group format during a walk [38, 39]. A focus group was felt appropriate for understanding the women's experiences as they were comfortable as a group, and the exchange of ideas was felt to be important [40].

The first author conducted interviews with both walk leaders and ran the focus group with the walk participants. The two interviews and the focus group lasted approximately 1 h each and were conducted in quiet areas of local coffee shops of the women's choosing. It is important to note that these were spaces in which the women and walk leaders were comfortable. They were conducted at quiet times of day with no possibility of the conversations being overheard; for example, one interview was held in an outside garden. The purpose was not to discuss personal health information, rather to gain insights into their experiences of the programme and how it could be improved. They were audio‐recorded, transcribed with any identifying information removed and the audio recording destroyed.

Data and fieldnotes were analysed deductively using a framework approach [41]. Data collection and initial analysis was conducted by SH listening carefully to the recordings. This was then discussed with WH to cross check the initial ideas. These discussions and cross checking aimed to ensure authenticity and rigour in our findings and interpretations [42].

## 3 Findings

Both iterations of the ‘Walk and Talk’ programme in 2020 and 2021 were observed. Both times, cancer was not discussed at all in the first week. Access to a dentist and a doctor was discussed during the second week (outside a medical practice). Confidence in having difficult conversations with a health professional, breast self‐examination, cancer screening and symptom awareness were all discussed at some point in the walks. All six participants on the second walk programme were white and only one was not British (European). They were aged between 23 and 58 years. One had a disability which meant that she walked slowly and had poor vision and hearing on one side. Both walk leaders are White British women. Our fieldnotes showed that walks typically lasted 45–60 min and between four and six women attended. There was good interchange of conversation between the women, and women changed positions in the group to chat to others. The disabilities of a new member were accommodated with women moving to speak on her unaffected side and slowing their pace. Although they started and finished at the centre, women would text if they would be late and would join the route. Sometimes women left early, for example, if the route was on the way to another appointment or nearer their home. As such, there was informality and inclusion in the delivery of the programme.

### 3.1 Interviews With Walk Leaders

#### 3.1.1 The Acceptability of ‘Walk and Talk’ to the Cancer Champion Walk Leaders

Both walk leaders had many years of experience delivering training and support to vulnerable women. Examples included modules around domestic abuse, confidence, wellbeing and sexual health. Neither had delivered cancer prevention programmes before. Neither was asked during the interview about their personal life or cancer‐related experiences, as this was not felt appropriate, but the organisation promotes itself as based on lived experience with peer‐supported programmes and that women feel they have ‘something in common’ with their support workers. Both women were white. Walk Leader 1 (WL1) was in her 30 s and WL2 in her early 40 s.

#### 3.1.2 The Delivery of the Programme

Walk Leader 1 (WL1) reflected on the training she received for the delivery of health information on the walks saying:

I just think if you make it very clear with anybody else you are training that it can be as relaxed as you want it to be and literally just drip feed and it, it doesn't have to be, you know, a stop and sit down and talk about this. The conversation goes wherever it goes. And if somebody starts talking about smears [cervical smear test], but that's not till week four and we're on week two, it doesn't matter.

(WL1)

We're happy to carry on and do it more informally, like one lady was talking about having a coil fitted and then obviously I kind of got into that conversation of having a smear done. I do feel like it's quite easy to incorporate this now.

(WL1)

The walk leaders were very attuned to the women's needs and incorporated this experience into the delivery and advice for future walks:

Some weeks you just might not get anything done because they might have had an absolute mental breakdown about something else, and that is just what we will focus on, focusing on them and what their needs are on that day.

(WL1)

The walk leaders also reflected on the style of delivery, empowerment, trust and the women's responses.

I thought it would be a bit more closed up, so I was very surprised how open people were. I didn't feel like I was teaching anything. I just felt like I was making a passing comment and that was enough and then it was like they were talking for 15 minutes. You plant the seed, and the conversation goes where it needs to go. It's not like I am saying you need to do this, or you need to do that. So, I think that works really well because I didn't want to be someone preaching, ‘you need to go to the doctors’.

(WL1)

Some people have learnt from experience that there's no point mentioning anything because they're not going to get listened to. And that's the thing, isn't it? Once you then find someone who will listen to you. It kind of gives you that bit more confidence.

(WL2)

One of the aims of the programme was to empower women to seek out medical help as our previous paper [33] demonstrated that women with complex social and mental health needs often felt let down by statutory services. This affected how likely they were to seek help for physical health problems. Women had little awareness of the NHS cancer pathway. These frustrations were voiced by WL2.

Pre‐Covid it was exactly the same. People couldn't get appointments with their doctors, they were made to feel like they were imagining their symptoms when actually all they wanted was a face‐to‐face appointment with the doctor, and it's the same now and they still can't get it unless I ring up or someone else who's supporting them rings up and asks for an appointment.

(WL2)

Both walk leaders reflected that the informality of delivering information on a walk, rather than in a classroom where people are expected to talk, and also the lack of eye contact, helped women feel more comfortable.

There has always been a lot of respect within the groups once they start those walks and when they're not in that classroom situation—when you get a chance to understand someone better away from behind a table basically.

(WL2)

Maybe it's because you're not giving eye contact and you know there's not that pressure that somebody's gotta talk. You know it just felt like there wasn't as much pressure than if you're kinda sat down looking at each other.

(WL1)

#### 3.1.3 Confidence in Delivering the Programme

WL1 reflected:

I felt like it was quite a lot of pressure on me. But it just flowed a lot easier than I thought it would with the ladies talking about their experiences.

(WL1)

We don't want people to look at us and be like oh who are they? What are they doing and think we just want it to be a kind of more peer support. We're just guiding in the walk really. I'm there to listen and to have a chat. We don't want it to be like I'm the teacher and you're the student kind of things. We want it to all feel equal I suppose.

(WL1)

#### 3.1.4 Practical Insights

The walk leaders had a very person‐centred approach to the design of their walks. For example, they were conscious of the design of the walking routes, due to restrictions some women may have as part of their community sentencing, or spaces where they may feel threatened.

Obviously managing the areas that you go, you have to be kept wary of the of routes where we're going to bump into people that may cause issues or that could trigger other things.

(WL2)

Both walk leaders had previously led group walks. During the training, we talked about what a good walk ‘looked and felt like’. Whilst these walks were promoted as a series of health walks with a focus on cancer prevention, they were also encouraged during the training to do themed walks, for example International Women's Day and utilise the natural environment around them. One of the walk leaders was particularly interested in history. She used a bridge and the site of an old jail to make parallels between how women were victimised as witches in the past and modern‐day shaming of women.

Actually, a lot of the history is around health anyway, isn't it? You look at old hospitals and things like that. Wherever you go there's history around health and unhealthy stuff. You know like the graves from the plague, there's always that kind of stuff which can be tied in.

(WL2)

During the training, we talked about how women talk about their intimate body parts and how this might be discussed. For example, we used the resources of CoppaFeel (https://coppafeel.org/) on breast awareness and self‐examination. One walk leader had also done further research, for example, into slang terms, and was aware of follow‐up so that women were not let down.

I also think it's important to know the basics and to know resources of where people would go if they do need some support and regardless of whether that's help or a personal situation, if we don't action it (with their permission obviously), then that's just another service that's let them down.

(WL2)

### 3.2 Findings From the Focus Group With Programme Participants

We invited all six participants from the second ‘Walk and Talk’ to a focus group. Four attended; two said they were busy on the day. We offered a follow‐up focus group or individual interviews but neither responded.

#### 3.2.1 The Acceptability of the Programme

We were keen to understand whether this format was a safe environment and acceptable to women as a way of receiving health information and talking about their concerns. Women had clearly picked up on the intention for the walks to feel informal and to discuss topics that were potentially sensitive. As with the walk leaders, a sense of informality and the positive difference between a classroom and delivery on a walk was noted.

I think people don't realise the simplest of things that people worry about and people might think you're worrying about nothing, but it is OK to worry and talk about these things. I don't know if anyone else finds this, but I get very anxious about things like this and talking to a doctor can be daunting, you feel like an idiot, like they will tell you there is nothing to worry about. Some doctors can be good, but some doctors aren't great when they see something simple because obviously, they see people with other things, but in your mind it is important.

(P4)

We had things we didn't expect to talk about, but people felt comfortable and so they spoke about things. It has been really informal, hasn't it, a conversation really, and then it is where the conversation has gone.

(P2)

If you were sitting down, it would have been like a medical review, like a list or something. So, I liked the informality.

(P3)

If you come to a normal course or something, you couldn't say I've got a smear test next week or a lump in my boob, what can I do about it, but with this group it felt like you could talk about those things.

(P2)

You don't want someone telling you, ‘don't forget to do this’ on a walk, you just want people to know [the Walk Leaders] are there on the walk and that they are approachable.

(P4)

#### 3.2.2 Trust and Inclusion

Vitally, our findings point to the important role of the Cancer Champion Walk Leader in creating a trusted environment and empowering women to make informed choices. The women felt safe to talk about, often intimate, health problems and were open to receiving heath information. Trust and inclusion are particularly important among women who have been let down in previous trusted and intimate relationships and by state services.

It's one of those things where you feel comfy, you know each other, and we all feel safe as a group we know we have got each other's backs.

(P1)

When you go out with your social support workers, the worst thing is people wearing lanyards. It is embarrassing sometimes because you might see people and they don't know you are struggling, and they see that and might start asking questions. I noticed that on this walk that didn't happen.

(P4)

#### 3.2.3 Help‐Seeking Behaviours

Participants appeared to express greater confidence in seeking help and greater awareness of symptoms and cancer screening.

Speaking in a group, others would say, you are better to get it checked out. That extra little push might be all you need…. Yes, some people would be like, I don't want to waste my doctor's time, but speaking in a group you get that little push that you need to go and get seen.

(P1 and P2)

I think when you join the walks it gives the women an opening to talk about what is going on and maybe we don't realise how it is done, but it is nice to have that bit of info there [information about cancer screening and symptom awareness].

(P1)

I think a lot of women wouldn't talk about their health until it is brought up in a forum where someone else talks about something and they think, actually, I could have something to say about this. Otherwise, they will just stay at home twiddling their thumbs and asking Dr Google.

(P1)

## 4 Discussion

Recent systematic reviews demonstrated that victims of intimate partner violence (including psychological abuse) are more likely than other women to delay screening or not attend screening for cancer [43] and that building trust is an important determinant in cervical cancer screening in under‐served women [44]. This study was conducted with women with a similar profile at high risk of cancer. Shoulder to shoulder: Walk and Talk was a co‐produced programme of cancer prevention themed health walks led by trained Cancer Champion Walk Leaders underpinned by the principles of motivational interviewing to support positive change [35]. The aim was to work with women, in their space, to develop a novel and sustainable intervention to enable the opportunity to have conversations about cancer screening, symptom awareness and help‐seeking behaviours.

Our findings suggest that it is safe, acceptable and practicable to deliver health information in a nontraditional way during group walks. Our Cancer Champion Walk Leaders appeared to be comfortable in delivering health information about cancer symptoms, cancer screening, the importance of early diagnosis in cancer and empowering women to have confidence in communicating with health professionals. Our focus group with women who had attended the programme suggested that they trusted the programme, that they felt it was a safe spacet and that there was some evidence of help‐seeking behaviours.

A recent review on health literacy [45] demonstrated the need to improve both the quality and source of communication, for example, by developing frontline professional skills and support, to enable meaningful engagement, and by modifying the context in which health communication occurs. We suggest that delivery of a community‐based intervention tailored for a high‐risk population such as was developed in our ‘Walk and Talk’ programme is an example of this. We also note that this review demonstrated that health literacy interventions with community (nonclinical) populations are not yet as common in the published literature as those for clinical populations [45]. Our research contributes towards this field by taking an asset‐based approach (which works with available assets such as existing community organisations and works with their strengths—in our case the pre‐existing group‐based work the walk leaders did with women). We worked with a community organisation supporting women with high health needs to co‐produce and deliver an intervention that was practical, relevant and acceptable to them.

A recent meta‐ethnography of qualitative studies on nonparticipation in colorectal cancer screening identified the complexity of nonparticipation. It suggests that this does not always result from active refusal but may be due to ambivalence, postponement, practical barriers or lack of support from healthcare professionals [46]. We specifically talked about ambivalence to changing health‐related behaviours [35]. In the intervention development workshops, women helped us to understand how they felt conflicted due to competing priorities in their often complex lives [33]. This became integral to the training with the walk leaders so that they were able to acknowledge these difficulties, encourage women to be kind to themselves, identify what mattered to them and empower them to make positive changes to their health and wellbeing.

A study on cancer symptom awareness in England pointed to embarrassment, such as changes in a woman's breasts, and a lack of confidence to talk to a doctor as barriers to seeking help [16]. Our findings point to the important role of finding the appropriate language. For example, our walk leaders used a popular website, and one used an urban dictionary, to find ways of talking about intimate body parts; the women agreed how these would be talked about. Women also role‐modelled conversations and made suggestions to each other to empower them to feel confident in speaking to health professionals about their needs.

Nutbeam and Lloyd [45] suggest that empowering people to act requires a fundamentally different approach to traditional health communication such as using more interactive and adaptable communication methods. By using a walking group, we sought to pilot and evaluate an innovative method: not only provide the opportunity to impart information but also to increase capability for positive change [24]. Importantly this information was developed by women for women, in their context [47]. Our findings point to the crucial importance of the person‐centred skills of the Cancer Champion Walk Leaders. For example, in adapting the programme and making minor adjustments to each walk so that the needs of the women were appropriately accounted for and trust was built to enable talk about change. This approach stands in marked contrast to many established communication models based on changing specific knowledge, attitudes and behaviours [45].

Finally, we note how time‐consuming intervention co‐production is, especially with groups underrepresented in research. This study was built on trusted relationships between the lead researcher and the community organisation. We believe that this vital relationship‐building enabled a trusted and authentic research environment to facilitate the inclusion of a woefully under‐researched group. From this, we were able to co‐produce an intervention in their space, on their terms, that met their needs and wishes.

A statement of the contribution that this work makes to the field of cancer prevention for high risk women and health literary can be found in File S3.

## 5 Strengths and Limitations

This study has strengths and limitations. A major strength is the careful development and co‐production of the programme using an asset‐based approach, working with an existing community organisation with trusted relationships and directly informed by our previous work [33]. This study demonstrated the high cancer risk, complex needs and high need for cancer prevention interventions in women who have suffered domestic abuse or are in the criminal justice system. Most importantly, ‘Shoulder to Shoulder: Walk and Talk’ was co‐produced with women with lived experience of domestic abuse and experience in the criminal justice system (custodial and noncustodial sentences) and piloted with the same population living in areas of high socioeconomic deprivation. We recognise that this is a very small evaluation. However, we consider it is vital that the women's views are validated at this early stage to enable optimisation by ourselves or others for further testing, as suggested by Skivington et al. [47] in the development of complex interventions.

A limitation is that all participants were white, which to a degree reflects the geographical area in which the study was conducted (East of England). As our study suggests, careful tailoring of the intervention to women in a particular circumstance is vital for trust to enable conversations on taboo and stigmatised subjects. We therefore recommend that this programme needs further tailoring and piloting if it was used with women from a different high‐risk population. The walking groups were already pre‐formed in the women's centre which we suggest enabled trust to be built quickly, but the creation and recruitment to such groups warrants further research.

Finally, the first author has had a trusted relationship with the community organisation for several years. We accept that this relationship building introduces a bias despite conscious reflexivity. We feel this is counter balanced by the authentic nature of our research that was done in a highly collaborative way, rather than research ‘done on’ people.

## 6 Conclusions

Although health literacy is not a panacea for reducing disparities in cancer prevention, our findings show that cancer prevention for high‐risk groups can be delivered in a personalised, novel and tailored way by creating the opportunity for supportive conversations about behaviour change in an informal way in a walking group. We carefully co‐produced the programme of walks with women, using scenarios and quotes that were authentic vignettes which came directly from the women's lived experience. This enabled women to talk about change on their own terms. Our findings indicate that this approach was acceptable with some evidence of women feeling empowered to make informed decisions about their health. We recommend that future cancer prevention interventions for underrepresented groups use an asset‐based approach to increase reach and sustainability.

## Author Contributions

**Sarah Hanson:** conceptualisation, investigation, funding acquisition, writing–original draft, methodology, writing–review and editing, formal analysis, project administration, data curation, resources. **Wendy Hardeman:** writing–review and editing, conceptualisation, methodology.

## Conflicts of Interest

The authors declare no conflicts of interest.

## Supporting information

Supporting information

Supporting information

Supporting information

Supporting information

## Data Availability

The data that support the findings of this study are available from the corresponding author (S.H.) upon reasonable request. This includes access to the full protocol.
