# Supplementary material for: The Co‐Production, Pilot and Qualitative Evaluation of a Cancer Prevention Programme With High‐Risk Women Delivered on Group Walks by Cancer Champions: Shoulder to Shoulder, Walk and Talk
Source: Health Expect. 2024 Aug 8;27(4):e14175. doi: 10.1111/hex.14175 (PMC11306970; doi:10.1111/hex.14175)
Supplement: Supplementary file 1 — Supporting information [file HEX-27-e14175-s003.docx]

**Topic guide – sample questions**

Focus groups with the women

What is your overall view of the walk and talk? Suggested probes: What are your views on getting health information in this way? What did you like? What did you not like? What might you do differently since attending them? Is there anything that has stuck in your mind since? Probe for examples such as getting health information in general, about behaviour change, knowledge about specific facts about cancer, screening, symptom awareness, help-seeking behaviours.

Discussions as a group on the ‘Walk and Talks’: how comfortable were you? How could we make it more comfortable / easier to talk? Or do you think it is not appropriate to discuss such things on a walk? How was it different to a general group walk?

The walk leader is not an expert in cancer but has had some training in helping women to feel more comfortable to talk about seeking help (such as seeing their doctor with symptoms and getting screened for cancer). Do you have any comments about how the walks were led? Was there anything you didn’t like? Was there anything that you particularly liked?

Generally. This study is a very small pilot to enable the study team to learn. Please can you give your suggestions so that we can improve the Walk and Talk programme.

Anything else to add?

Interviews with the walk leaders

What is your overall view of the ‘Walk and Talk’ programme? Probe for specific examples. Do you have a view on how effective it was?

How did you structure the series of walks? Probe: For example, how did you space out the information. Did you do a different topic each walk or mix it up a bit?

In your view, how receptive were the women? What factors do you think helped to make it receptive? If you have any examples of reticence / reluctance /difficulties, how did you handle these?

You had some basic training on behaviour change, leading a walk, facilitation skills, cancer symptoms, screening programmes and help-seeking. What in the training was helpful and relevant? What was not helpful / not relevant? What more training / support do you need to deliver the ‘Walk and Talk’ programme well?

Anything else to add?
