# Supplementary figures and images for: The Co‐Production, Pilot and Qualitative Evaluation of a Cancer Prevention Programme With High‐Risk Women Delivered on Group Walks by Cancer Champions: Shoulder to Shoulder, Walk and Talk
Source: Health Expect. 2024 Aug 8;27(4):e14175. doi: 10.1111/hex.14175 (PMC11306970; doi:10.1111/hex.14175)

**Outline walks training programme**


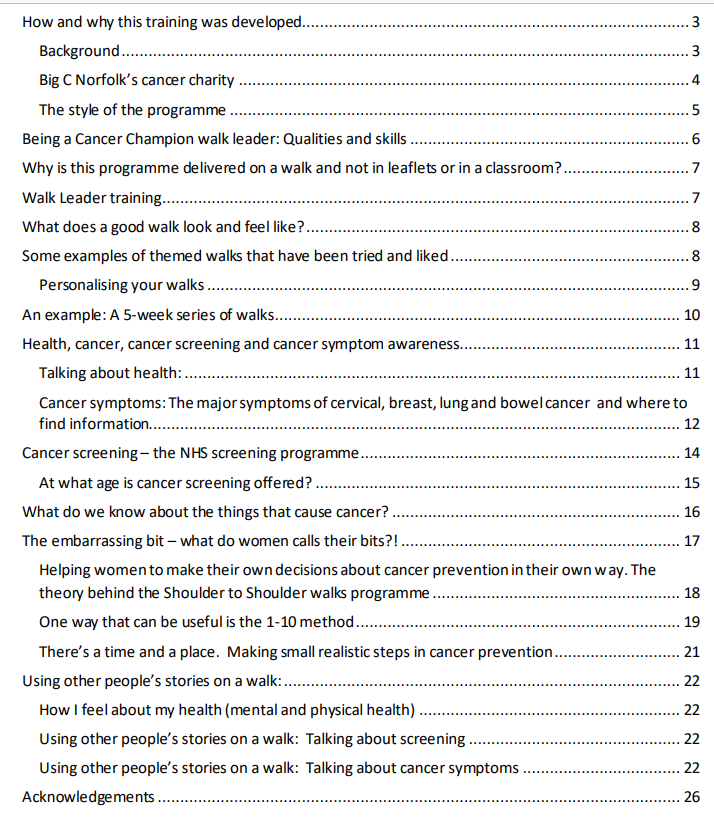

Supplement: Supplementary file 2 — Supporting information [file HEX-27-e14175-s001.docx]
