# Supplementary material for: The Co‐Production, Pilot and Qualitative Evaluation of a Cancer Prevention Programme With High‐Risk Women Delivered on Group Walks by Cancer Champions: Shoulder to Shoulder, Walk and Talk
Source: Health Expect. 2024 Aug 8;27(4):e14175. doi: 10.1111/hex.14175 (PMC11306970; doi:10.1111/hex.14175)
Supplement: Supplementary file 3 — Supporting information [file HEX-27-e14175-s002.docx]

**Statement of Contribution**

What is already known about this subject?

Women in the criminal justice system and women who have been subject to domestic abuse are at high risk of cancer.

Cancer prevention programmes often fail to reach high risk women due to personal circumstance & poor health literacy.

Women at high risk are underrepresented in health research and intervention designs rarely include their input.

What does this study add?

A co-produced programme delivered during group walks enabled supportive conversations about cancer prevention.

A programme designed by women in their space appeared acceptable, empowering and increased confidence to seek help.

The potential for asset based cancer prevention programmes to increase reach and sustainability.
