# Supplementary material for: The Co‐Production, Pilot and Qualitative Evaluation of a Cancer Prevention Programme With High‐Risk Women Delivered on Group Walks by Cancer Champions: Shoulder to Shoulder, Walk and Talk
Source: Health Expect. 2024 Aug 8;27(4):e14175. doi: 10.1111/hex.14175 (PMC11306970; doi:10.1111/hex.14175)
Supplement: Supplementary file 4 — Supporting information [file HEX-27-e14175-s004.docx]

**Data collection sheet: Shoulder to shoulder: Walk and Talk (StS)**

| **Location**: **Walk number**:  **Date**:  **Start time. Finish time. Length of time out on the walk**:  **Weather**:  **Brief description of the walk circuit**: | **No of participants**:  **Approx. age of participants**:  **Equipment/clothes**:  **Register completed**:  **Consent and questionnaires completed**: |
| --- | --- |
| **Comments and reflections on**: | |
| **Completion of the questionnaire (*if applicable*)**: | **Any comments by participants about the walking group / being part of the research**: |
| **Conversations covered during this walk: (*including approximate time spent on each*)**   - ***General*** *(e.g Walk Leader led topics, e.g. debt advice, local issues and topics introduced by the walkers)* - ***Health*** *(in its widest sense, e.g. to include diet and mental health, advice given between participants)* - ***Specific*** *(StS)* | |
| **How cancer prevention topics introduced: (*example phrases, participant responses*)** | |
| **How walks organised: (*e.g. who goes at the front, dyads of conversation, people switching positions*)** | |
| **Atmosphere and dynamic**: | **Researcher presence: *(‘nicely irrelevant’*)** |
| **What happened before the walks**: | **What happened at the end of the walks**: |
| **Thoughts for next iteration of StS: (*e.g. the use of the cue cards / vignettes*)** | **Any other ideas and thoughts**: |
